# Temperature induced structural distortion and their effect on the physical properties of La_0.6_Sr_0.4_Mn_0.8_Co_0.2_O_3_ nanoparticles

**DOI:** 10.1038/s41598-025-14101-0

**Published:** 2025-08-12

**Authors:** M. A. Awad, Abdalrahman M. Rayan, I. A. Abdel-Latif, Mahrous R. Ahmed

**Affiliations:** 1 Physics Department, Faculty of Science, Sohag University, Sohag, 82524 Egypt; 2 Department of Reactor Physics, NRC, Egyptian Atomic Energy Authority, Cairo, Egypt

**Keywords:** LSMCO, Rietveld refinements, Jahn-Teller distortion, Halder-Wagner, FTIR, Semiconducting, superparamagnetic-like behavior, Physics, Applied physics, Condensed-matter physics

## Abstract

The current study presents an unprecedented detailed investigation of the structural, magnetic, electrical, molecular, morphological and compositional analysis of the La_0.6_Sr_0.4_Mn_0.8_Co_0.2_O_3_ (LSMCO) nanoparticles synthesized by the sol-gel method and calcinated at 650, 1000, and 1100 °C. Rietveld refinements and FTIR analysis, confirmed the formation of perovskite structure, where Rietveld output was used to trace the effect of calcination temperature on the overall structural properties. The Jahn-Teller distortion in LSMCO was inferred from the rhombohedral distortion, cooperative octahedral tilting/canting, electron distribution density, and shifts in Mn–O bond absorbance. The Halder-Wagner model and Scherrer equation were used for accurate crystallite size calculations. Thanks to structural analysis, the magnetic behavior and conduction mechanism in the LSMCO nanostructure were well explained. Magnetic analysis demonstrated superparamagnetic-like behavior at 1100 °C. Electrical measurements in the temperature range showed semiconducting behavior (298–473 K), where dual activation energies were identified. The activation energy at low temperatures (0.02–0.11 eV) was ascribed to bond length and bandwidth changes, whereas the activation energy at high temperatures (0.138–0.24 eV) was ascribed to polaron-mediated conduction and to Jahn-Teller distortion. These findings demonstrate the potential of using LSMCO nanoparticles in applications such as medical fields and in advanced magnetic storage systems.

## Introduction

In the past 50 years, the successive interest in the preparation of magnetic nanomaterials has been a hot topic due to the variety of magnetic applications^1–5^. Perovskites lanthanum strontium manganite (LSMO) are rich materials used for various applications due to the excellent magnetic and electrical characteristics that are dependent on preparation conditions^6^. The distinctive perovskite structure and the replacement of La³⁺ by some Sr²⁺ ions induced the mixed-valence states of Mn⁴⁺ and Mn³⁺ that enhanced the electrical conductivity, polaron hopping, and magnetic properties^7^. Researcher efforts have been dedicated to exploring the suitable techniques for LSMO preparation, among them sol-gel^1^, hydrothermal^8^, co-precipitation^9^, combustion method^10^, solid state method^3^ etc. The sol-gel method is characterized by high purity, small particle size, large product yield, and uniform nanostructure at low synthesis temperature.

The introduction of Co ions into the LSMO crystal structure is significantly modified the magnetic interactions. Where cobalt incorporation disrupts the double exchange interaction and increase the superexchange interaction^11^. As a result, the material exhibits reduced saturation magnetization, increased antiferromagnetic behavior, and enhanced short-range magnetic ordering. This in turn generates a disordered glassy system through random distribution of cobalt ions in the perovskite structure that generates a state known as ferromagnetic clusters, antiferromagnetic clusters, and superparamagnetic-like free clusters^12^. These mixed phenomena were observed in magnetic systems having crystallite sizes ranging within the nanometer scale (10–150 nm) that propelled them in various applications such as spintronic^12^ magnetic storage devices^13^, biomedical fields^14^ and in cancer treatment^15^ etc.

Various reports have verified the influence of Co doping on the magnetic properties of LSMO nanostructures. Phuc et al.^16^ confirmed that the ferromagnetism of LSMO is deteriorated by Co doping, where the superexchange interaction is predominated even at a low Co ratio. Chen et al.^12^ proved that the transport and magnetic properties of LSMO are greatly affected by Co concentration. The samples exhibit ferromagnetism with metallic conducting behavior at very low or high concentrations. Where the intermediate doping came to the sample in a mixed magnetic state in which a glassy state is observed.

The main objective of our study is to provide detailed structural and functional analysis of La_0.6_Sr_0.4_Mn_0.8_Co_0.2_O_3_ nanoparticles synthesized by the sol-gel method and calcinated at 650, 1000, and 1100 °C. Our work fills the gap between temperature-induced structural changes such as canting angles, bandwidth, bond lengths, bond angles, electron distribution density, and physical properties, including magnetic, electrical, molecular, and morphological properties. Furthermore, our manuscript provides comprehensive Rietveld refined parameters, such as atomic positions, thermal factors, and tilting/canting angles, which offer a valuable dataset for future researchers studying perovskite-based compositions. Unlike previous studies focused on varying Co ratio on the properties of LSMO nanoparticles, our study focused on a fixed Co doping level of x = 0.2 in La_0.6_Sr_0.4_Mn_0.8_Co_0.2_O_3_. Where increasing calcination temperature to 1100 °C enhanced the Co incorporation into the perovskite lattice, that disrupts the double exchange interactions and strengthened the antiferromagnetic interaction. This process led to the formation of superparamagnetic-like behavior, which suggested their potential applications in medical or magnetic targeting fields. To the best of our knowledge, such a comprehensive dataset has not been previously reported for this specific composition.

## Experimental procedures

### Sample preparation

**Fig. 1:**
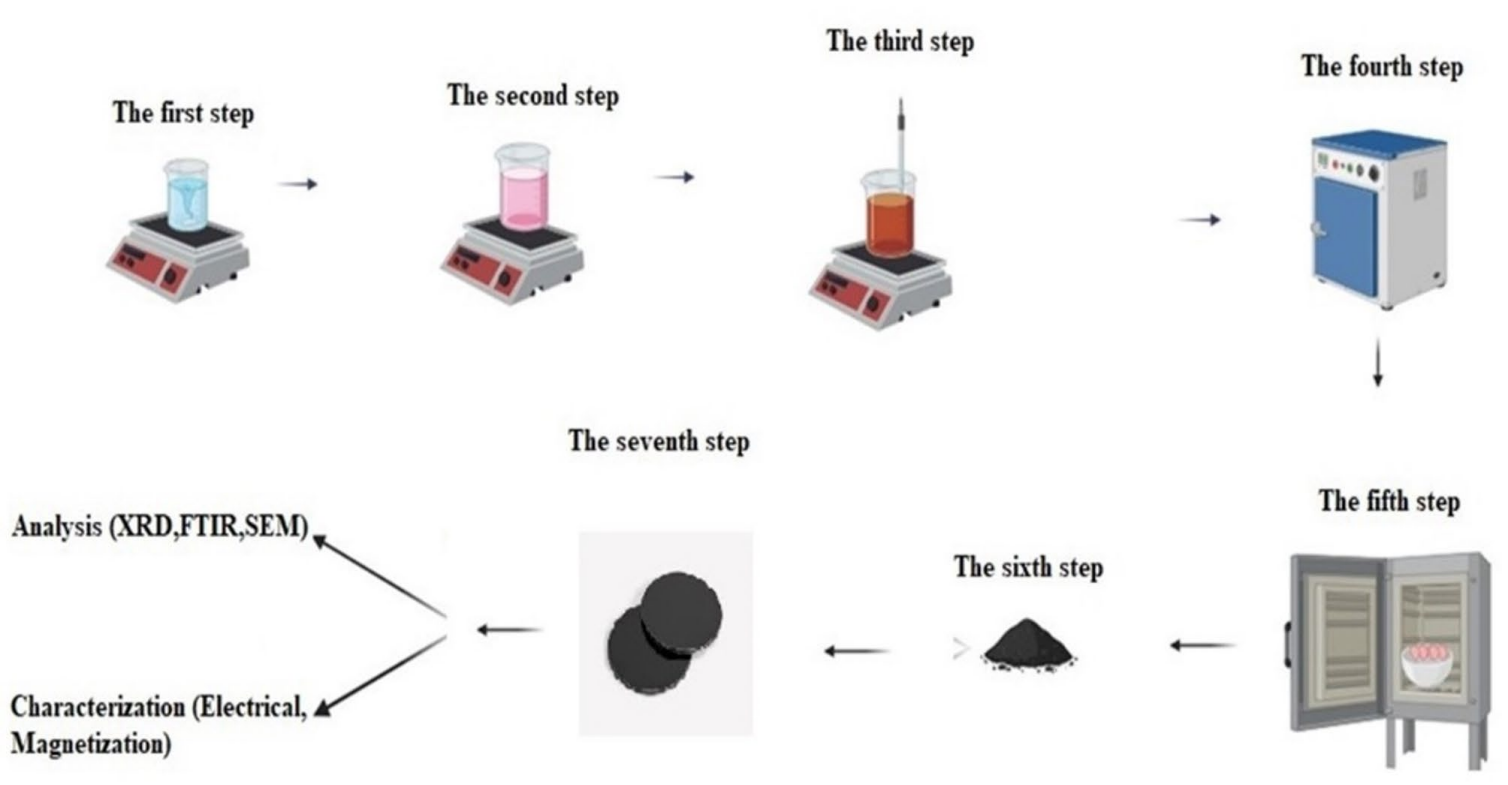
The steps to prepare LSMCO nanoparticles at different calcination temperature (650 °C (a), 1000 °C (b) and 1100 °C (c).

La_0.6_Sr_0.4_Mn_0.8_Co_0.2_O_3_ NPs were synthesized by the sol-gel method. The chemicals were lanthanum nitrate hexahydrate (LaNO_3_.6H_2_O), strontium nitrate (Sr (NO_3_)_2_) and manganese nitrate (Mn (NO_3_)_2_) that were purchased from Sigma Aldrich with high purity (99.99%), cobalt nitrate hexahydrate (Co (NO_3_)_2_.6H_2_O) was purchased from Alpha chemical (purity 99%). Different steps were carried out to achieve the stoichiometric formula of La_0.6_Sr_0.4_Mn_0.8_Co_0.2_O_3,_ as indicated in Fig. 1. In the first step, the 0.01 mol of citric acid (template for nanoparticle growth) was dissolved in 100 mL of deionized water and then stirred for 30 min at 80 °C. In the second step, the appropriate weights of the aforementioned precursors were added, where a continuous stirring at 80 °C for 30 min was carried out until a clear light pink solution was obtained. In the third step, the ammonia solution was gradually added and stirred for 4 h at 80 °C thus raising the PH value to 9, where the color of the solution was gradually changed from clear light pink to light brown. In the fourth step, the reaction was left to cool down for 2.5 h, then a drying process was carried out at 105 °C for 36 h in a normal atmosphere. In the fifth step, a calcination process was carried out separately at 650, 1000, and 1100 °C in an electrical muffle furnace for 8 h, where the product was subsequently ground in agate mortar for 30 min in the sixth step. In the seventh and final step, the ground powder is compressed at room temperature in the form of pellets of diameter 1 cm diameter using a Carver hydraulic press under a pressure of 150 bars for one min for electrical study.

### Sample characterization

Many analytics were carried out in order to verify the prepared samples, such as XRD using a Bruker D8-Advanced X-ray diffractometer with CuK_α_ radiation of 1.54060 Å. Rietveld refinement has been investigated using FullProf software to verify the crystal structure and calculate the structural parameters. The samples calcinated at 650 and 1000 °C were refined as mixed phases, whereas the 1100 °C sample was single phase refined. Thompson-Cox-Hastings pseudo-Voigt (axial divergence asymmetry) was the function used to refine the peak shape and full width at half maximum (FWHM). Mapping of electron distribution density per unit cell was also calculated using the G-Fourier software. The simulation of the crystal structure was carried out by Vesta software^17^. The surface morphology was performed using SEM (JEOL, Japan). The magnetic measurement was carried out using a vibrating sample magnetometer (VSM-7410, Lakeshore). The energy dispersive X- ray analysis (EDAX), the unite attached to SEM, was carried out to investigate the elemental composition and stoichiometric ratio of the LSMCO nanoparticles. The electrical properties were measured using a Keithley 175 A auto-ranging multimeter. FTIR spectra were recorded on a Bruker Platinum ATR spectrometer (FT-IR Alpha) using the attenuated total reflection (ATR) technique. The spectra are presented in wavenumbers (cm^− 1^), spanning a range of 4000 –400 cm^− 1^ with 4 cm^− 1^ resolutions.

To facilitate the reader, the La_0.6_Sr_0.4_Mn_0.8_Co_0.2_O_3_ was referred to as LSMCO, and samples calcinated at 650, 1000, and 1100 °C were referred to as samples S1, S2, and S3, respectively.

## Results and discussion

### Structural analysis

**Fig. 2:**
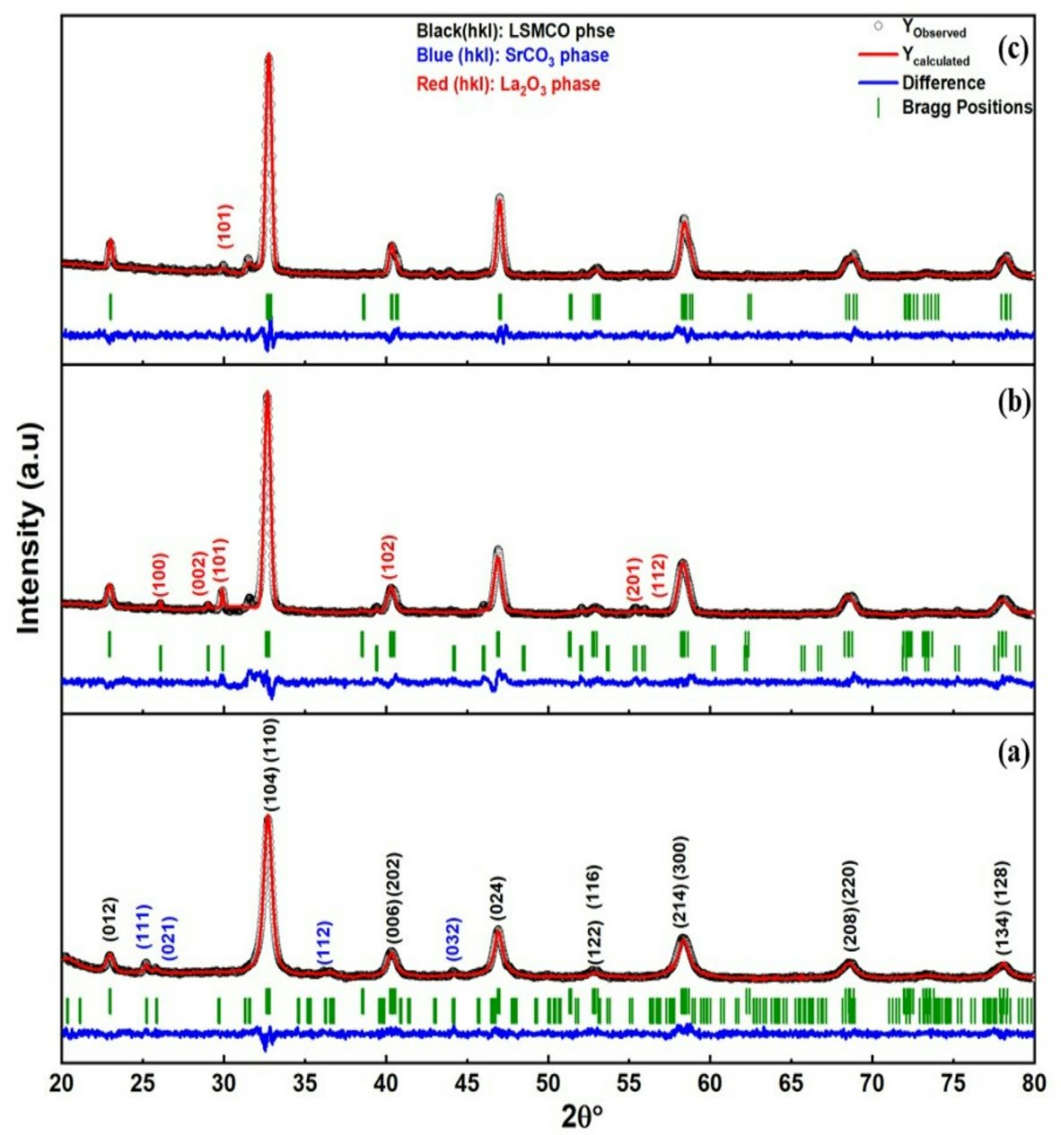
Rietveld refinement of LSMCO nanoparticles at different calcination temperature (650 °C (a), 1000 °C (b) and 1100 °C (c).

**Fig. 3:**
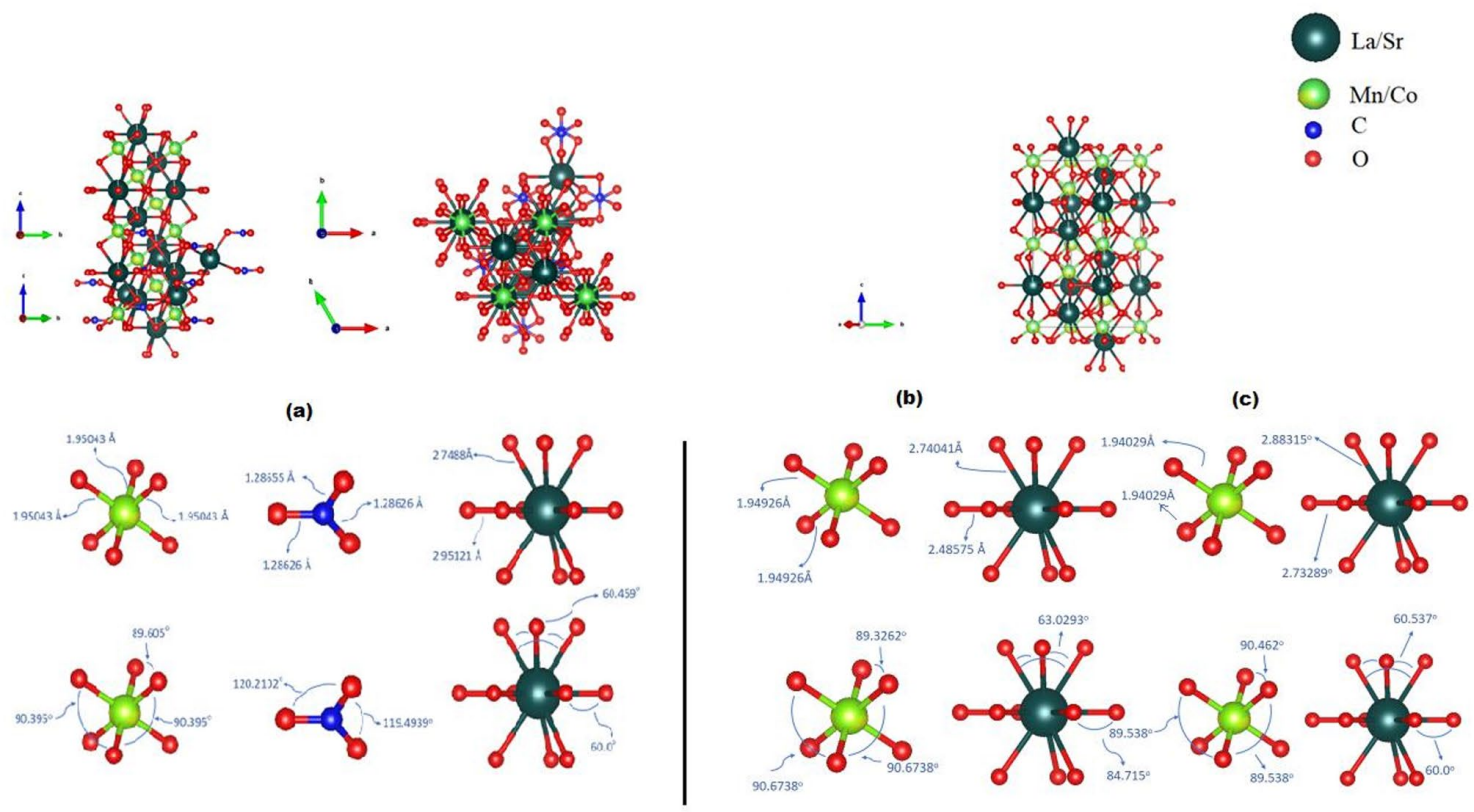
The crystal structure and the changes in bonds length - angles of LSMCO nanoparticles at different calcination temperature (650 °C (a), 1000 °C (b) and 1100 °C (c).

The XRD of LSMCO nanoparticles at different calcination temperatures is indicated with the black line (observed) of Fig. 2. Where a polycrystalline nature is observed and confirmed with increasing calcination temperature (650, 1000, and 1100 °C). By comparing the diffracted peaks with JCPDS files (Fig. 2a), it is discerned that they perfectly match the hexagonal phase of LSMCO (ICSD Collection Code: 050711) and the orthorhombic phase of SrCO_3_ (COD 9013802). Increasing calcination temperature to 1000 °C (Fig. 2b) promoted the phase separation of the hexagonal La_2_O_3_ phase (COD 1531452), in addition to the main LSMCO phase (COD 4002478). As seen from Fig. 2c, although increasing calcination temperature to 1100 °C has no direct effect on increasing crystallinity, the La_2_O_3_ phase tends to be attenuated in addition to the decrease in FWHM, which is related to the increase in nucleation and growth rate with elevating the calcination temperature^18^. The calculated Rietveld refinement (red line in Fig. 2) shows good fitting with experimental data ( black line), where the χ^2^ value in Table 1 is an indicator of goodness of fit^19^. Table 1 summarizes the refined parameters of the main LSMCO phase, where the unit cell volume is irregularly changed with calcination temperature. The reason relied on the incorporation of SrCO_3_ with lattice parameters of (a = 5.1006, b = 8.4107, c = 6.0181 Å) and La_2_O_3_ with lattice parameters of a = b = 3.9442, and c = 6.1499 Å into the LSMCO unit cell. Table 2 summarized atom positions and thermal parameters of the main LSMCO phase at different calcination temperatures. It is noticed that most of the atom’s positions are changed as a result of heat treatment and due to SrCO_3_ plus La_2_O_3_ incorporation into the LSMCO unit cell. The thermal parameters give an indication of atoms’ vibrations within the unit cell in all directions. From Table 2, it is evidenced that the LSMCO phase appeared to be isotropic for all calcinated samples, whereas SrCO_3_ appeared to be anisotropic^20,21^. Figure 3 (a, b and c) describes the crystallography simulation using Vesta software, where the red balls represent the oxygen atoms that are distributed octahedrally around the manganese and cobalt atoms (phosphorous balls). This occurred due to the approximation of ionic radii of both manganese and cobalt ions^21^ that permitted the successful replacement. The strontium atoms (olive balls) also exchanged their positions with lanthanum atoms, whereas carbon atoms popped up through the blue color. The second phase of SrCO_3_ is embedded within the hexagonal LSMCO unit cell at 650 °C, while La_2_O_3_ is incorporated within the hexagonal unit cell at 1000 °C (Fig. 3b). Here the Mn ions existed in two oxidation states, Mn^3+^ (3d^4^ configurations) and Mn^4+^ (3d^3^ configurations)^22^. On the other hand, cobalt existed in trivalent Co^3+^and tetravalent Co^4+^oxidation states^22,23^.

**Table 1:** The refined parameters of LSMCO nanoparticles at different calcination temperature (650 °C, 1000 °C and 1100 °C).

Calcination temperature (ºC)	Crystal system	Space group	Lattice parameters (Å)	Volume (Å^3^)	χ^2^
650	Hexagonal	R -3 c	a = b = 5.4850(13), c = 13.3584(22)	348.0529	1.32
1000	Hexagonal	R -3 c	a = b = 5.487586(86), c = 13.377548(48)	348.8750	2.64
1100	Hexagonal	R -3 c	a = b = 5.489066(66), c = 13.333860(60)	347.9243	1.53

**Table 2:** The atom positions and thermal parameter of LSMCO nanoparticles at different calcination temperatures (650 °C, 1000 °C and 1100 °C).

Sample		Atoms	Atomic positions			Thermal parameter			
			x	y	z	-			
650 °C	LSMCO	La	0.0	0.0	0.25	1.79876			
		Sr	0.0	0.0	0.25	1.79876			
		Mn	0.0	0.0	0.0	1.44787			
		Co	0.0	0.0	0.0	1.44787			
		O	0.45397	0.0	0.25	0.71690			
	SrCO_3_	Sr	0.25000	0.41619	0.75678	Anisotropic	B11: -0.02402	B22: -0.00809	B33: -0.00586
		C	0.25000	0.75873	0.91453	Anisotropic	B11: 0.22089	B22: -0.15304	B33: -0.00586
		O1	0.25000	0.91118	0.90499	Anisotropic	B11: 0.90868	B22: -0.06476	B33: 0.22491
		O2	0.46785	0.68183	0.91390	Anisotropic	B11: 0.17609	B22: 0.00578	B33: -0.15600
1000 °C		La	0.54609	0.0	0.25	2.20902			
		Sr	0.0	0.0	0.0	0.95834			
		Mn	0.0	0.0	0.25	1.92504			
		Co	0.0	0.0	0.25	1.92504			
		O	0.0	0.0	0.25	1.92504			
1100 °C		La	0.52525	0	0.25	1.23865			
		Sr	0.0	0.0	0.0	1.15082			
		Mn	0.0	0.0	0.25	0.89457			
		Co	0.0	0.0	0.25	0.89457			
		O	0.0	0.0	0.25	0.89457			

**Fig. 4:**
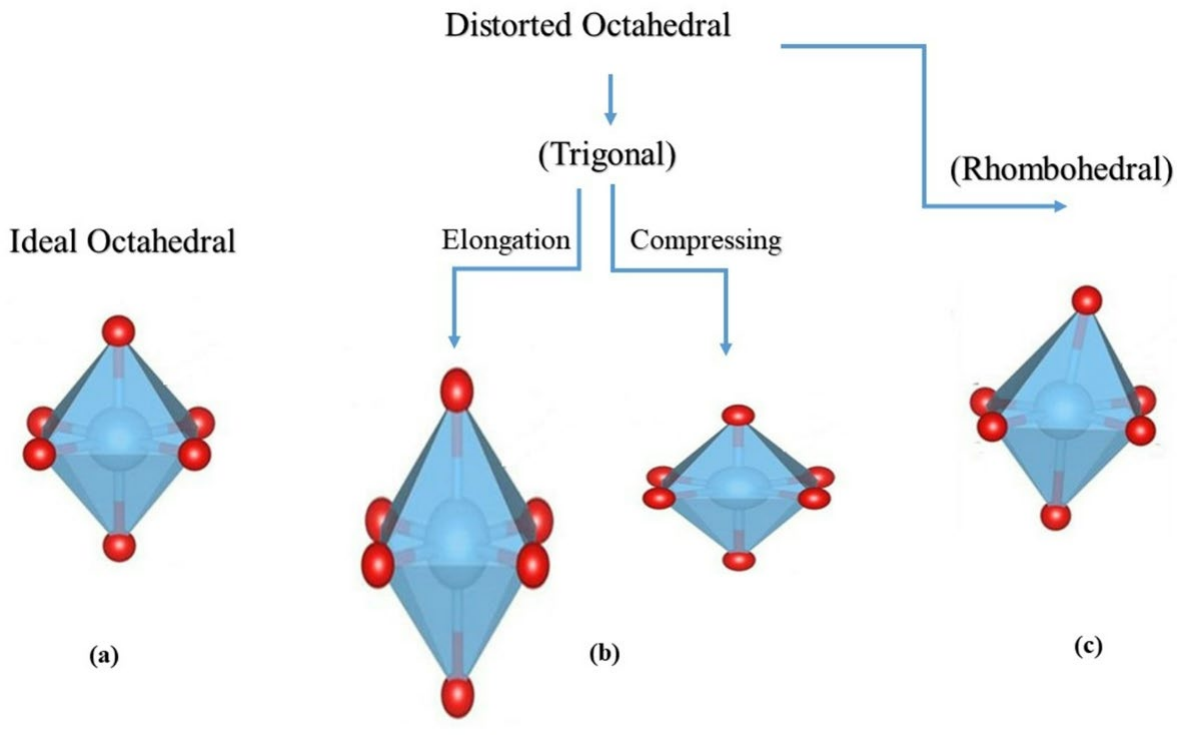
The trigonal and rhombohedral distortion of LSMCO nanoparticles.

The Jahn-Teller distortion within the LSMCO structure was evaluated by calculating the local distortion in the MnO6 octahedra. As illustrated in Fig. 3, the uniform Mn-O bond lengths at constant temperature suggest the absence of trigonal distortion, where a homogeneous compression distortion appears in the octahedron with increasing calcination temperature. In contrast, the deviation from the ideal O-Mn-O bond angle of 90° indicates the presence of rhombic (bond angle) distortion. This rhombic (bond angle) distortion was quantified using the bond angle variance equation^24–26^:

1 $${\sigma ^{\text{2}}}=\frac{1}{{m - 1}}\mathop \sum \limits_{{i=1}}^{m} {\left( {{\xi _i} - ~{{\xi }_o}} \right)^2}$$

where σ^2^ is the bond angle variance, m is the number of O-Mn-O bond angles (12 for an octahedron), ${\xi _i}~$ is the measured bond angle, and ${{\xi }_o}$ is the angle of a perfect octahedron (90°). The values of bond angle variance (σ^2^ for S1, S2, and S3 are 0.0284, 0.0831, and 0.0381, respectively. These results indicate the presence of rhombohedral distortion in the MnO_6_ octahedra that influences the electrical and magnetic properties of LSMCO nanoparticles. This occurs in order to lower the internal energy within the unit cell through decreasing the symmetry without changing the crystal structure^27,28^ and this is confirmed in Table 2 through changing atom positions. Figure 4 explains the disparity between trigonal and rhombohedral distortion. Worth noting, measurement of bond length and angles is very important when studying the electronic and various physical properties of the perovskite structure^29^.

**Fig. 5:**
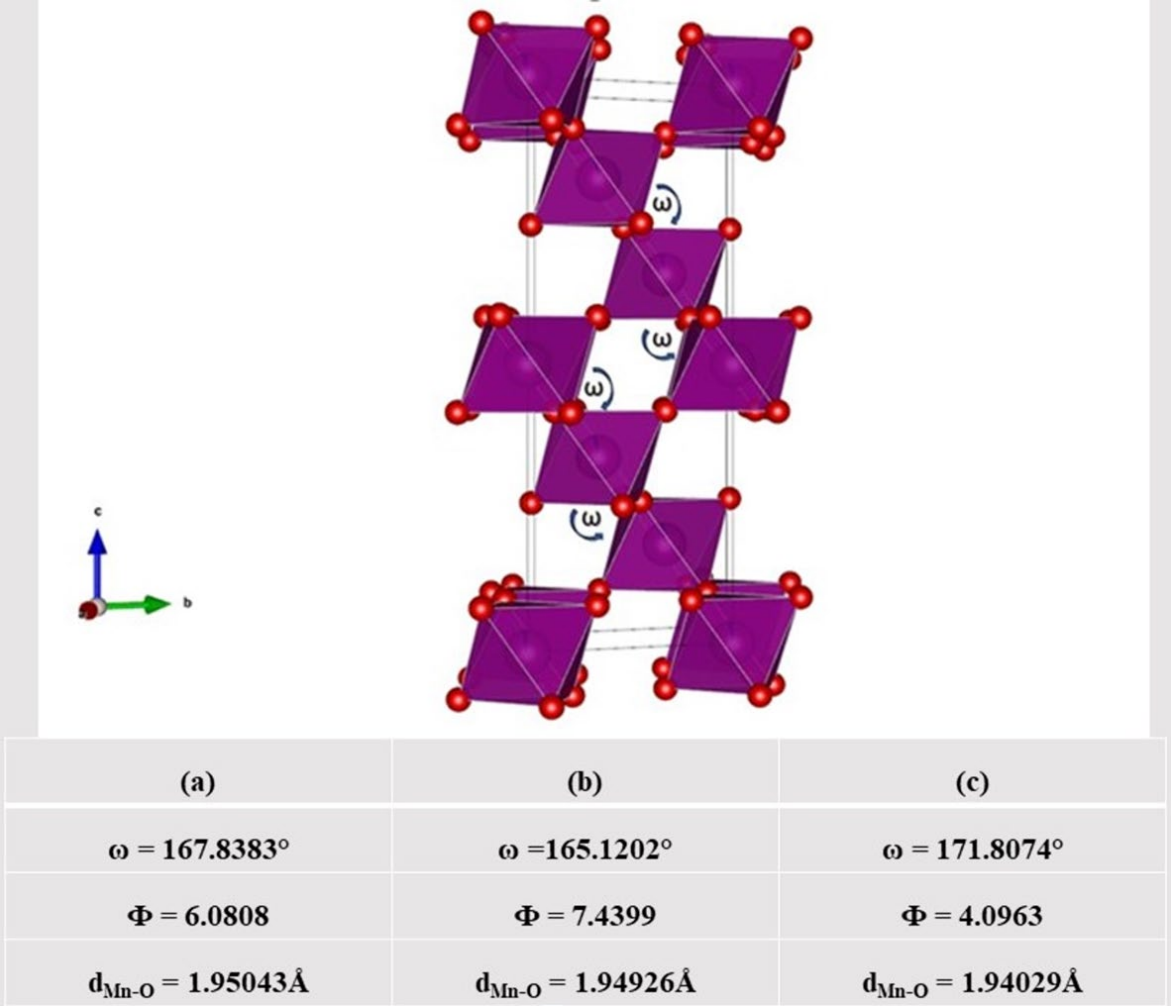
The canting angle(ω) and tilting angle (Φ) of LSMCO nanoparticles at different calcination temperature (650 °C (a), 1000 °C (b) and 1100 °C (c).

To trace the cooperative Jahn-Teller distortion between octahedral when temperature is increased, Fig. 5 (a, b and c) describes the simulated results for the three calcined samples. The tilting angle (Φ) is calculated using the equation Φ = (180 - ω)/2^30^, where (ω) is the canting angle between the two (Mn/Co - O - Mn/Co) octahedra as indicated in Fig. 5. It is noticed that there are changes in both the tilting and the canting angles with increasing calcination temperature.

**Fig. 6:**
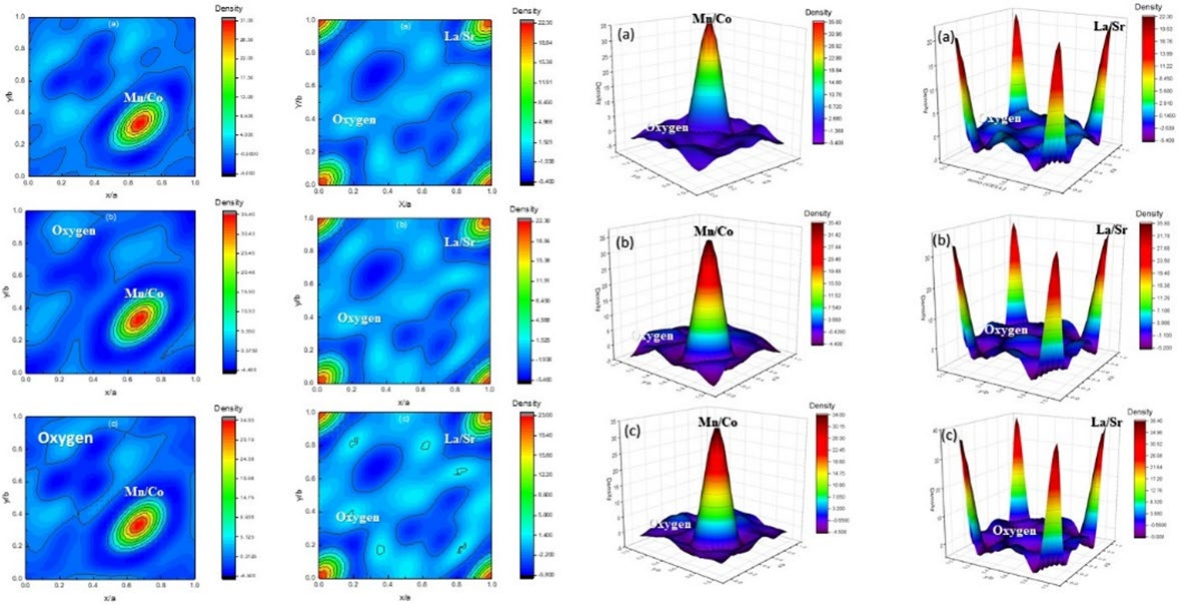
The electron distribution density of LSMCO nanoparticles at different calcination temperature (650 °C (a), 1000 °C (b) and 1100 °C (c).

Figure 6 describes the electron distribution density inside the unit cell of LSMCO nanoparticles at different calcination temperatures. These calculations are investigated via the G-Fourier software using Rietveld output, where atom positions and intensities of electron wavefunctions are indicated within the unit cell. The equation used to describe electrons’ distribution density ρ(r) in the unit cell^31^ is given by,

2 $$\rho \left( {\text{r}} \right){\text{ }}=\frac{1}{V}\mathop \sum \limits_{H} F\left( H \right).{\text{ }}{{\text{e}}^{[ - {\text{2}}}}^{{\pi {\text{i}}\left( {{\text{H}}.{\text{r}}} \right)]}}$$

where V is the volume of the unit cell, r is the position vector inside the unit cell and H is the reciprocal lattice vector^32^. It is evidenced from Fig. 6 that raising calcination temperature to 1100 °C decreases the charge density of Mn^+ 3^ ions (0.67 Å) as a result of Co^+ 3^ (0.65 Å) ion implantation^21^. This in turn decreases the covalent and ionic radii between cobalt-oxygen ions (d_Co−O_) compared with Mn-oxygen ions (d_Mn−O_), which in turn makes closed shell interaction and charge depletion in the mid-bond interaction^21^ as indicated in Fig. 3. On the other hand, the charge density of La/Sr increases with increasing calcination temperature. This is related to the complete replacement of La^+ 3^ ions (1.06 Å) with higher ionic radii Sr^+ 2^ ions (1.13 Å). The variation of charge density confirms the presence of Jahn-Teller distortion. Worth noting, studying electron distribution density is helpful for the interpretation of electrical and magnetic properties of the prepared samples^31^.

The crystallite size (D) was calculated using the Scherrer equation^33^ and the Halder-Wagner model^34^ after correction due to instrumental broadening. Where Scherrer’s formula is given through

3 $$D = \frac{K \lambda}{\beta \cos\theta}$$

in which β is the FWHM of the diffracted peaks, $\lambda$ is the wavelength of the used X-ray, K is the shape factor that depends on the diffractometer properties, and $\theta$ is the detector rotation half angle. The substitution in the Scherer equation results in an increase in crystallite size with increasing calcination temperature, as indicated in Table 3. The increase in crystallite size is correlated with the decrease in dislocation density as a result of increasing the nucleation and growth rates^18,35^.

**Table 3:** Crystallite’s size (D), strain (ε), retentivity, band width and activation energy of LSMCO nanoparticles at different calcination temperature (650 °C, 1000 °C and 1100 °C).

Calcination temperature (ºC)	D (nm) Scherrer	D (nm) Halder-Wagner	Retentivity (emu/gm)	Band width	Activation energy (eV)	
					E_a1_	E_a2_
650	22.2	14.934 ε = 0.0018391	-0.0102	0.509822	0.11 ± 0.0312	0.24 ± 0.03154
1000	73.9	42.79 ε = 0.0040153	-0.0232	0.508696	0.02 ± 0.04042	0.14 ± 0.01791
1100	88.8	59.982377 ε = 0.0026943	0.0051	0.514078	0.03 ± 0.02671	0.138 ± 0.03533

For more accurate crystallite size and strain calculations, the Halder-Wagner model^34^ was utilized. Notably, this approach assumes that peak broadening conforms to Voigt symmetric functions, comprising both Lorentzian and Gaussian functions. This underscores the significance of the Halder-Wagner model in such cases. The Halder-Wagner model is described by Eq. (4) below:

4 $$\left( {\frac{\beta }{{2\tan \theta }}} \right)^{2} = ~\frac{1}{4}~\frac{{k\lambda }}{D}~\frac{\beta }{{\tan \theta ~\sin \theta }} + (2\varepsilon )^{2}$$

**Fig. 7:**
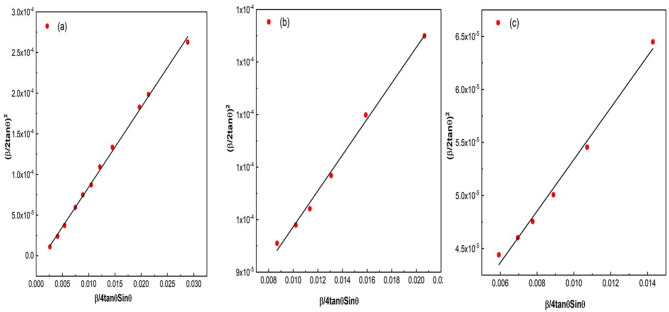
The Halder-Wagner model for calculating crystallites size of LSMCO nanoparticles at different calcination temperature (650 °C (a), 1000 °C (b) and 1100 °C (c).

The crystallite size and strain are estimated through the linear plot of (β/2tanθ)^2^ versus 1/4 β/(tanθ sinθ), where the crystallite size is deduced from the slope while the strain is determined from the intercept. Figure 7 illustrates the Halder-Wagner fitting^34^ for LSMCO nanoparticles in the three calcined samples. The resulting crystallite sizes and corresponding microstrains are summarized in Table 3. The negative intercept observed for sample (a) indicates compressive strain, suggesting that the crystallites have undergone shrinkage. In contrast, sample (b) exhibits a positive increase in strain, indicating tensile strain, which is consistent with the observed growth in crystallite size. In sample (c), which exhibits the highest crystallinity, the strain is lower, probably because of minimizing lattice defects in the crystal structure, leading to less microstrain.

### The magnetic and electrical studies

**Fig. 8:**
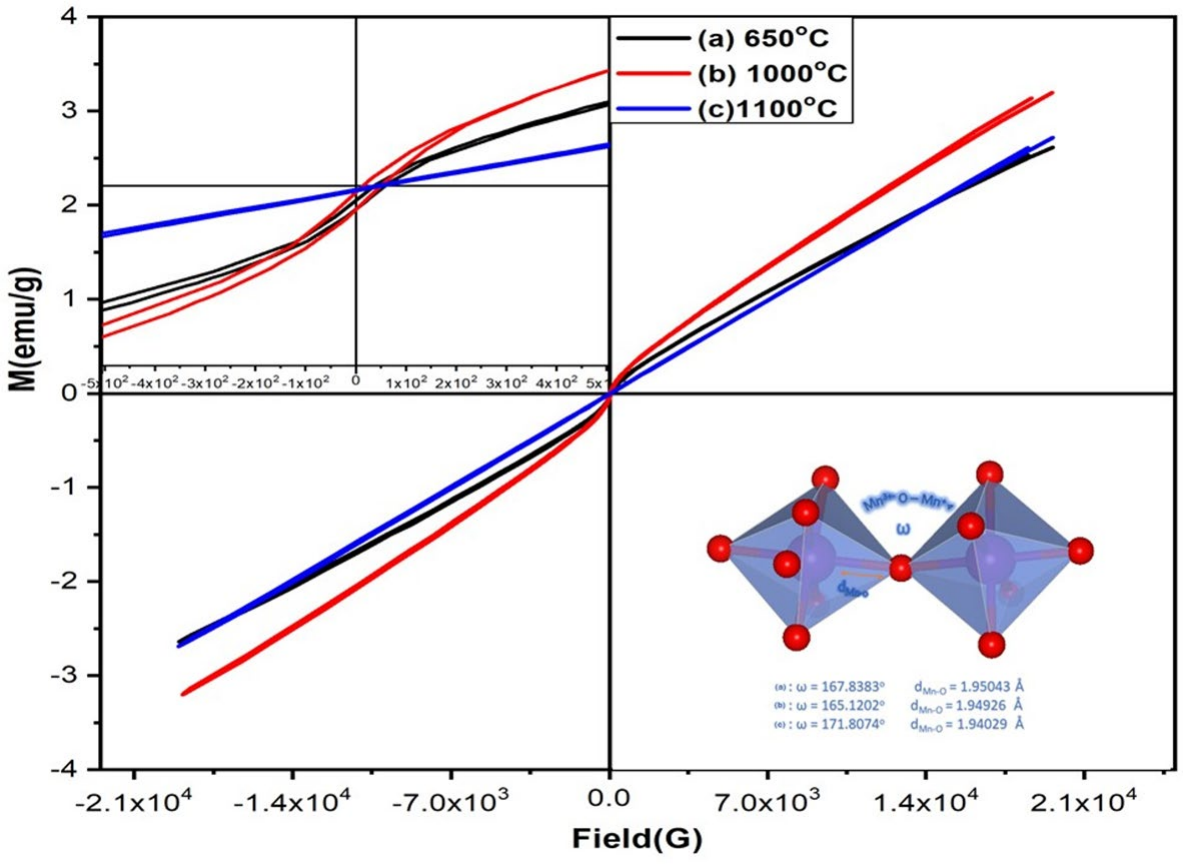
The magnetization versus magnetic field at room temperature for LSMCO nanoparticles at different calcination temperature (650 °C (a), 1000 °C (b) and 1100 °C (c).

In order to interpret the magnetic behavior of LSMCO nanoparticles at different calcination temperatures, it must be returned to the fact that the origin of magnetization in the LSMO perovskite structure originates from the coupling interaction between manganese and oxygen ions^36^. Where Mn^+ 4^ ions must have existed in addition to Mn^+ 3^ when La site was replaced by some Sr ions in LSMO unit cell^37^. Figure 8 represents the dependence of magnetization on applied magnetic field at room temperature. A mini-hysteresis-like behavior is observed at low magnetic field for S1 and S2 and is not prominent in S3. Clearly, there are two opposite competing interactions that take place when applying a magnetic field: the double exchange via electron hopping from the e_g_ orbital of Mn^+ 3^ to Mn^+ 4^ through O^2−^ ions while preserving the spin direction^37^ and the superexchange interaction induced by Co ions^22^. Increasing calcination temperature stimulates the Co ions to be substituted into the Mn position with their trivalent and tetravalent oxidation state (Co^3+^/Co^4+^)^37^. This provides an electronic mixture between double exchange and super exchange magnetic coupling^38^. The double exchange interaction is not greatly affected by the nanophase^38^ and takes place by electron transitions between Mn^3+^ - O^2−^ - Mn^4+^, Co^3+^ - O^2−^ - Mn^4+^ and Co^3+^ - O^2−^ - Co^4+^ ions of different oxidation states. The superexchange interaction occurred between ions of the same oxidation state, such as Co^3+^ - O^2−^ - Co^3+^ ions and Co^4+^ - O^2−^ - Co^4 +^^22^. The superexchange reduced the saturation magnetization, induced magnetic short-range ordering, and increased the antiferromagnetism^16,39^. It should be noted that in superexchange interaction the electrons are localized, on contrast to the double exchange interaction where the electrons are delocalized^40^. As seen from Fig. 8, the hysteresis loop is shifted from the origin as a result of exchange interaction^41^in which a competition occurred between the ferromagnetic and antiferromagnetic components when the particle size was reduced to the nanometer scale (less than a micrometer). The secondary phases of SrCO₃ and La₂O₃ have an impact on the magnetic behavior, particularly on the retentivity values shown in Table 3. Their presence is associated with the reduction in spin rotation when the magnetic field is reversed, thereby retaining some magnetization^36^. Consequently, these phases are crucial in modifying the material’s magnetic hysteresis properties.

The electron bandwidth (W)^38^ is determined to clarify the interaction type in LSMCO nanoparticles. It represents the distance at which the electron traveled between two adjacent cations and is given through,

5 $${\text{W }}=\frac{{\left( {\frac{{Cos\left( {\pi - ~\omega } \right)}}{2}} \right)}}{{{d_{Mn - O}}}}~~$$

where ω is the canting angle (twisting angle) between Mn^3+^ - O - Mn^4+^ and ${d_{{\text{Mn}} - {\text{O}}}}$ is the bond length between the Mn and O ions. Returning to the crystallographic data of Rietveld refinement, it is indicated that the canting angle (ω) is significantly varied with non-sequential change while bond lengths decreased with increasing calcination temperature. Substituting the values of canting angle (ω) and ${d_{Mn - O}}~$in Eq. (5) yields values of bandwidth that are listed in Table 3. The increase in bandwidth for S3 confirms the Co incorporation in the Mn position that impairs the electronic hopping and increases the super exchange interaction, which decreases magnetic properties^38^. It was previously reported that Co substitution in the Mn position increased the magnetic interaction between them and hence changed the electron spin direction, leading to the superexchange interaction^38^. The antiferromagnetism is also confirmed via canting angle, where its value detects the type of interaction. When ω is in the range from 120 to180°, a strong antiferromagnetic behavior occurs, which increases the superexchange^42^. It is concluded that the produced system is inhomogeneous and unsaturated and acted as superparamagnetic-like behavior^12,43^which was inferred from the sudden changes in magnetization when the magnetic field is reversed, especially in the S3 sample.

**Fig. 9:**
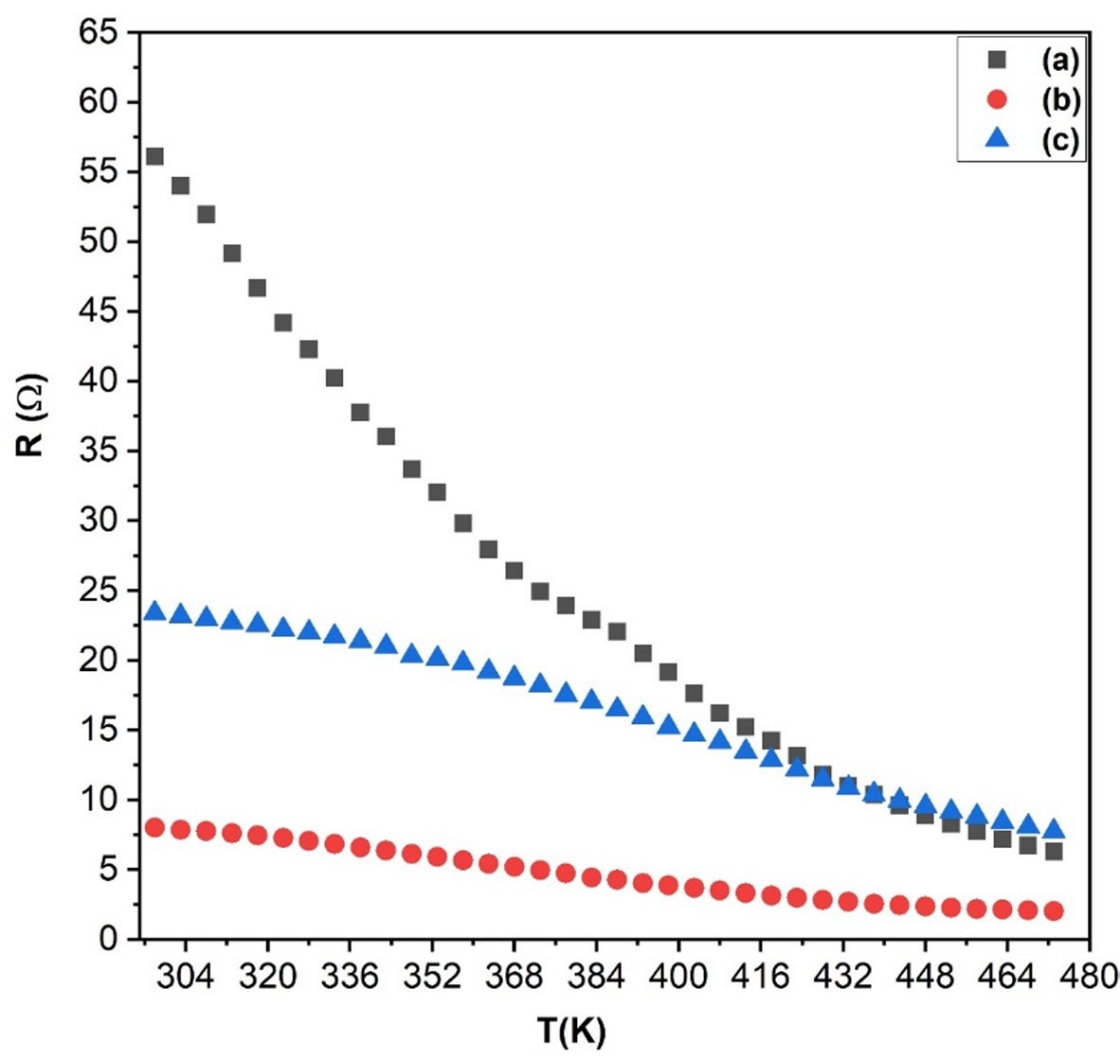
The electrical behavior of LSMCO nanoparticles at different calcination temperature (650 °C (a), 1000 °C (b) and 1100 °C (c).

To interpret the electrical behavior of LSMCO nanoparticles in the temperature range (298–463 K), Fig. 9 demonstrates the attitude. Semiconducting behavior is observed for the three calcinated samples. The disparity of resistivity at room temperature is caused by the preparation history, where phase separation in S1 confirms the incomplete substitution of Sr^2+^ ions, which in turn increases the electrical resistivity, acting as a trap of charge carriers^37^. A similar effect is observed with the La₂O₃ phase at 1000 °C. However, the complete incorporation of Sr^2+^ into the LSMCO lattice (S2) induces the formation of Mn^4+^ ions^37^which enhances the charge transfer between Mn^3+^ and Mn^4+^ ions, thereby reducing overall electrical resistivity. This improves the charge transport, diminishing the negative effects of La₂O₃ as a separate phase, as indicated in Fig. 9b. The rise in calcination temperature to 1100 °C increases oxygen desorption as well as Co^3+/4+^ ion substitution that increases the electron bandwidth (W), hence increasing the electrical resistivity. It was previously reported that oxygen desorption has a positive effect on increasing the electrical resistivity^40^.

**Fig. 10:**
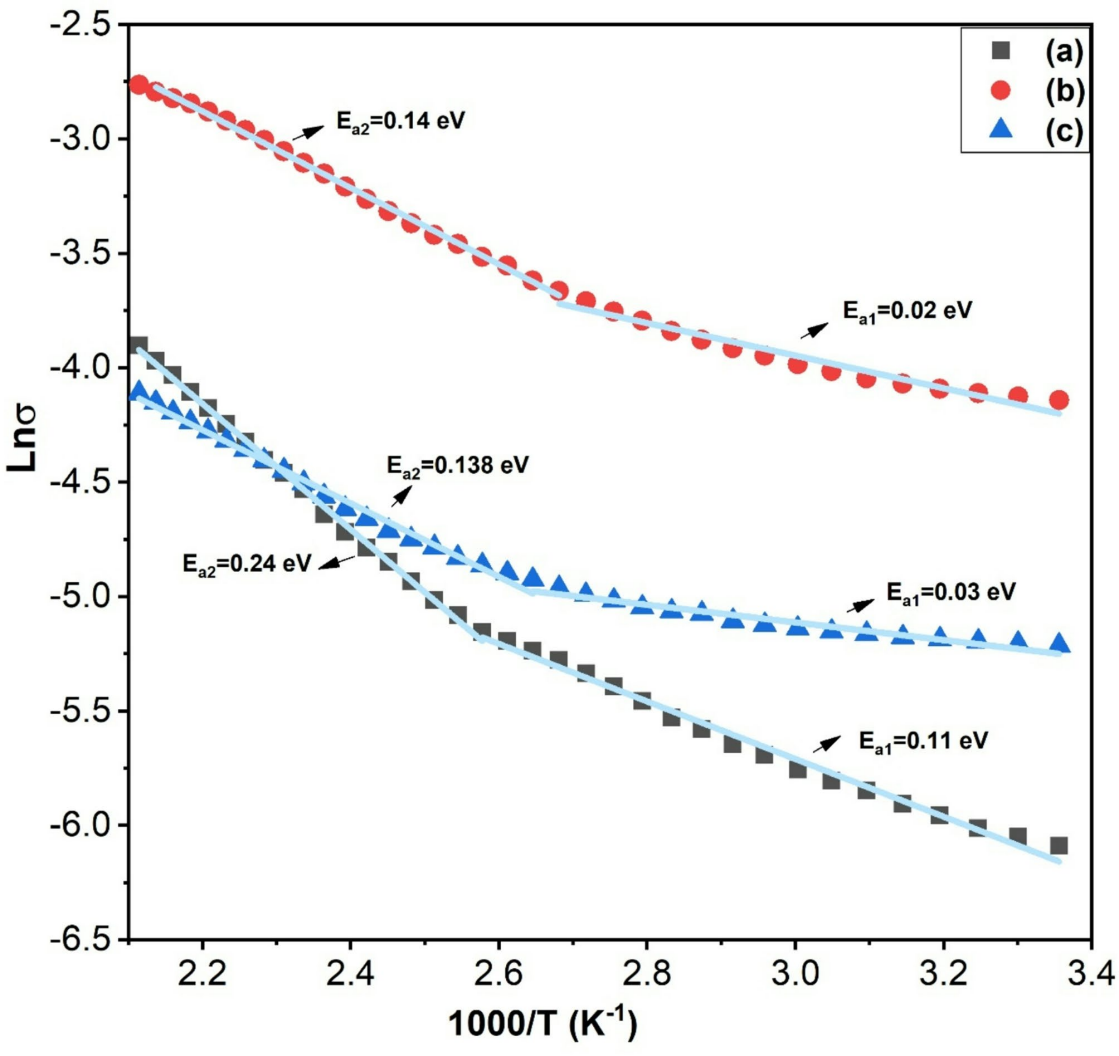
Lnσ versus 1000/T for LSMCO nanoparticles at different calcination temperature (650 °C (a), 1000 °C (b) and 1100 °C (c).

To understand the conduction mechanism in the LSMCO NPs, the activation energy (E_a_) is calculated by applying the Arrhenius relation given by

6 $$\sigma ~\left( T \right)=~~{\sigma _o}~{e^{\frac{{ - {E_a}}}{{{K_B}T}}}}$$

where σ(T) is the electrical conductivity, T is the temperature in Kelvin, and K_B_ is Boltzmann’s constant. Figure 10 depicts the plot of Lnσ versus 1000/T in which semiconductor behavior is observed. The Arrhenius fitting shows two activation energies for each calcinated temperature that confirm the transition between two conduction mechanisms. The activation energy in the low-temperature range (E_a1_) is best explained on the basis of electron bandwidth, where the activation energy is in synchronization with the changes in bandwidth. This study confirms that the activation energy in this range is governed by electron transition via double exchange interaction. Conversely, polaron hopping, phase separation, superexchange interaction, and particle size govern Ea_2_ in the high-temperature range. For S1, the large activation energy (E_a2_) is correlated to the localization of e_g_ electrons in Mn^3+^ which decreases the polaron hopping^44^in addition to phase separation acting as a trap for the free carriers^45^. The significant drop in activation energy in S2 is related to the addition of Sr^2+^, which boosts the number of charge carriers and creates hole polarons^44^. It is precedented from Rietveld calculation the existence of Jahn-Teller distortion that creates Jahn-Teller magnetic polarons^46^. The increase in crystallite size and the presence of a single phase in S3 are enough to decrease the activation energy (E_a2_) at high temperatures that are compensated by Co ion-induced superexchange and antiferromagnetism. Worth noting, the values of E_a2_ are close to those reported in the literature^47^.

### The morphological and molecular studies

**Fig. 11:**
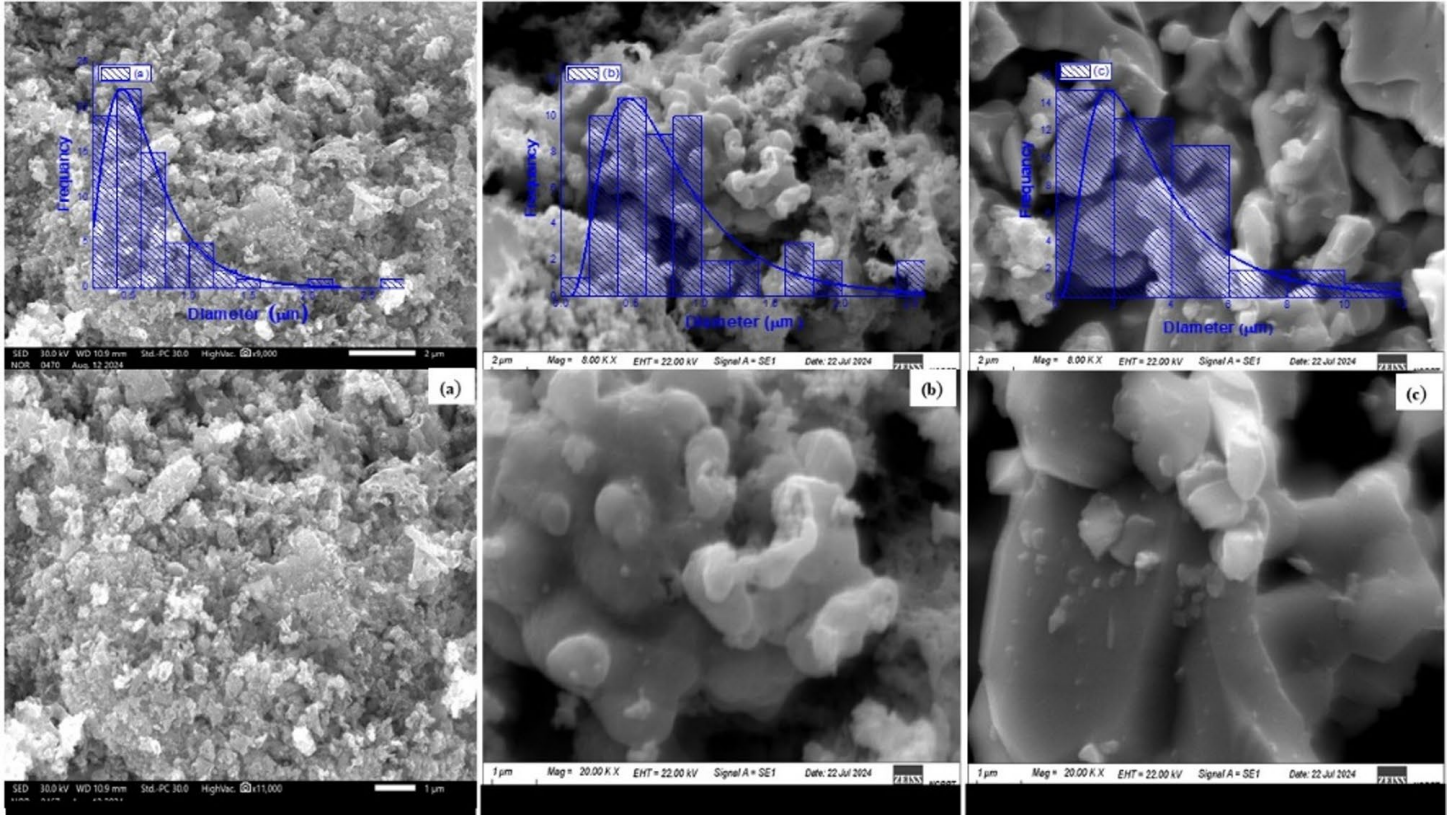
The surface morphology and particle size distribution of LSMCO nanoparticle at different calcination temperature (650 °C (a), 1000 °C (b) and 1100 °C (c).

Figure 11 reveals valuable insights into the surface morphology and particle size distribution of LSMCO nanoparticles. The figure shows clear proof that all samples create unevenly clumped nanoparticles, where particle size increases as the calcination temperature increases^18,35^. The discrepancy between the particle size from SEM images and the crystallite size determined by XRD is attributed to the agglomeration of particles induced by opposing magnetic dipoles^35,48,49^. This finding makes a significant contribution to our understanding of the properties and behavior of LSMCO nanoparticles.

**Fig. 12:**
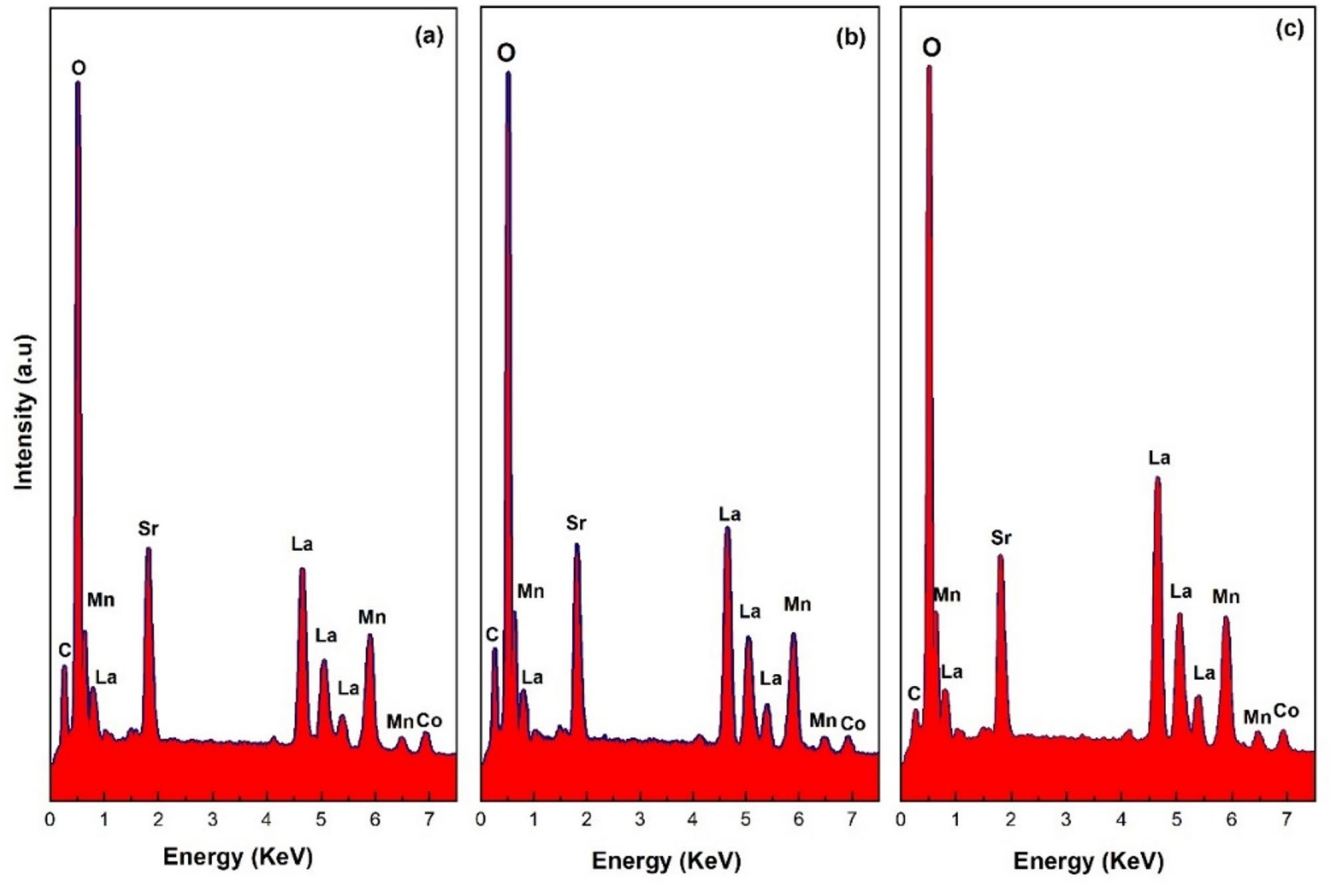
The qualitative EDAX analysis of LSMCO nanoparticle at different calcination temperature (650 °C (a), 1000 °C (b) and 1100 °C (c).

**Fig. 13:**
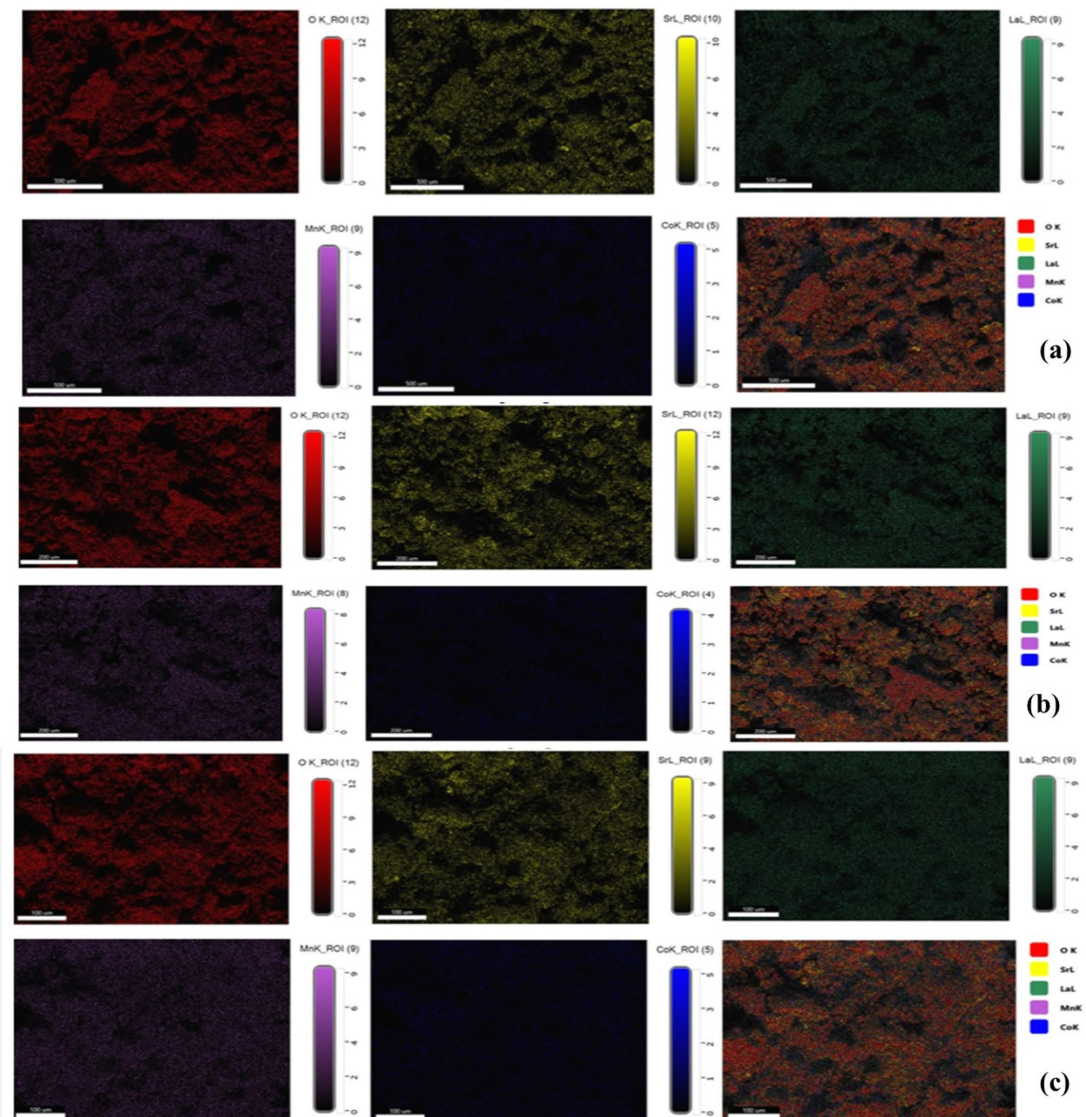
The elemental mapping of LSMCO nanoparticle at different calcination temperature (650 °C (a), 1000 °C (b) and 1100 °C (c).

To investigate the elemental composition of LSMCO nanoparticles, EDAX analysis was performed. The qualitative analysis shown in Fig. 12 reveals the presence of the characteristic X-ray peaks corresponding to La, Sr, Mn, Co, and O in all three calcinated samples^50,51^. The absence of any other elemental peaks indicates the high phase purity, where the weak carbon (C) signal originates from the SEM vacuum system contamination. The quantitative analysis of LSMCO NPs is summarized in Table 4. There are no significant changes in the element ratios with the increase in the calcination temperature. To clarify how much the elemental ratio matches the nominal stoichiometry, the normalized atomic percentages are taken based on La. Where it is noticed that the elemental ratios at 650, 100, and 1100 °C are La_0.6_Sr_0.25_Mn_0.63_Co_0.18_O_2.62,_ La_0.6_Sr_0.22_Mn_0.54_Co_0.11_O_2.22_ and La_0.6_Sr_0.17_Mn_0.51_Co_0.103_O_1.86,_ respectively, closing to the nominated stoichiometric composition of La_0.6_Sr_0.4_Mn_0.8_Co_0.2_O_3_. The elemental mapping (Fig. 13) was performed to evaluate the spatial distribution of the constituent element. The result shows that the La, Sr, Mn, Co, and O elements are uniformly distributed within the composition with no signs of separation into different phases.

**Table 4:** The chemical composition of LSMCO nanoparticles at different calcination temperature (650 °C (a), 1000 °C (b) and 1100 °C (c).

Element	Weight%	Atomic %	Net Int	Error
Sample (a)				
O	21.8	61.2	1296.9	8.7
Mn	18	14.7	531.9	4.5
Co	5.5	4.2	111.5	8.8
Sr	11.5	5.9	505.6	7.2
La	43.3	14	777.7	3.8
Sample (b)				
O	20.4	60.2	1307.6	8.7
Mn	17.7	14.6	537.1	4.6
Co	3.7	3	81.2	11.6
Sr	11	5.9	511.7	7.1
La	47.9	16.3	823.7	3.6
Sample (c)				
O	18.3	57.2	1363.5	8.7
Mn	17.2	15.7	620.8	4.4
Co	3.8	3.2	94.9	10.6
Sr	9.3	5.3	488.3	7.3
La	51.4	18.5	1132.5	3.5

**Fig. 14:**
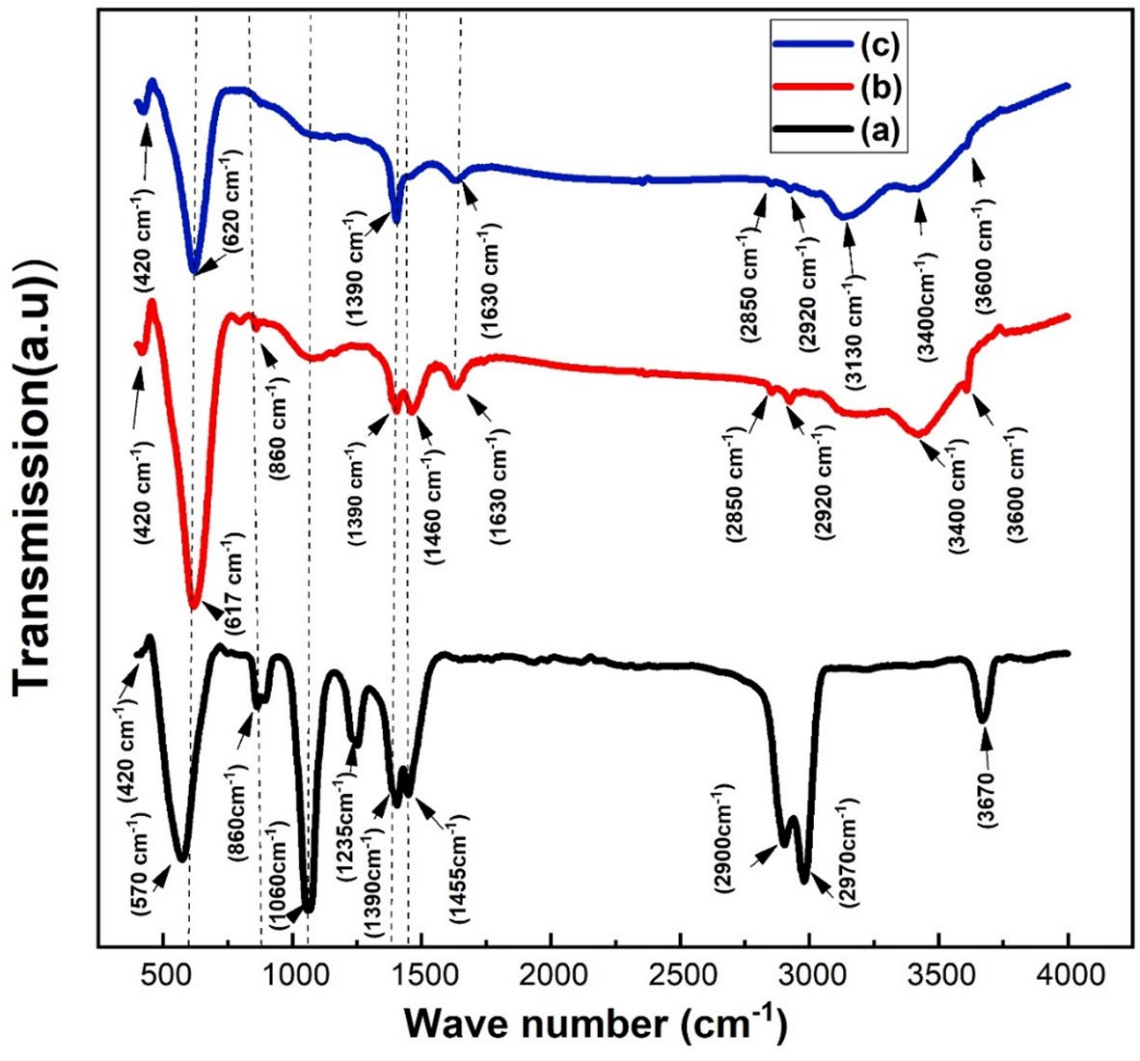
FTIR analysis of LSMCO nanoparticles at different calcination temperature (650 °C (a), 1000 °C (b) and 1100 °C (c).

The FTIR analysis shows the functional groups in LSMCO nanoparticles at different calcination temperatures (650, 1000, and 1100 °C). Figure 14 demonstrates the molecular spectra over the wavenumber range of 300–4000 cm^− 1^. It is clear that both S2 and S3 exhibit nearly identical behavior, with a slight shift due to heat treatment; in contrast, S1 shows extra absorption bands. Based on previous reports^52^, the octahedral MnO_6_ exhibits two IR-active modes out of its six possible vibration modes. Referring to Fig. 14, the formation of the manganite perovskite structure is confirmed by the presence of absorption bands around 400 and 600 cm^− 1^. The absorption band around 400 cm^− 1^ corresponds to the bending mode induced angle changes between Mn-O-Mn ions. On the other hand, the absorption band around 600 cm^− 1^ corresponds to the stretching mode-induced bond length changes between Mn-O-Mn and Mn-O ions^52^. These changes confirm the Jahn-Teller distortion due to heat treatment and Co^3+^ - Sr^2+^ incorporation, which in turn influences on the electrical and magnetic properties. The two absorption bands around 860 and 1060 cm^− 1^ in S1 are related to out-of-plane bending mode of the C-O bond in carbonate^53^where they are almost attenuated in S2 and S3. These findings are aligning with XRD, where the SrCO_3_ phase is disappearing in S2 and S3. The attenuation of the peak around 1460 cm^− 1^ indicates the carbonate decomposition^53^.The inward absorption band around 1235 cm^− 1^ corresponds to S1 only, which indicates the formation of the CH3-CH3-, CH3-NH_2_-, and CH3-O- band because polymerization occurred via citric acid and metal nitrate^54^. This band is disappearing at higher calcination temperatures in good finding with the disappearance of SrCO_3_ in XRD results. The absorption band around 1390 cm^− 1^ is originated from the bending vibrations of the N-O bonds, whereas the band around 1630 cm^− 1^ is assigned to the N-H bending vibrations^55^. In S1, all absorption bands around 2900 cm^− 1^, 2970 cm^− 1^ and 3670 cm^− 1^ correspond to the O-H bands due to the water absorbed from the atmosphere, where these bands are gradually attenuated and shifted towards lower wavenumbers with increasing calcination temperature (S2 and S3).

**Fig. 15:**
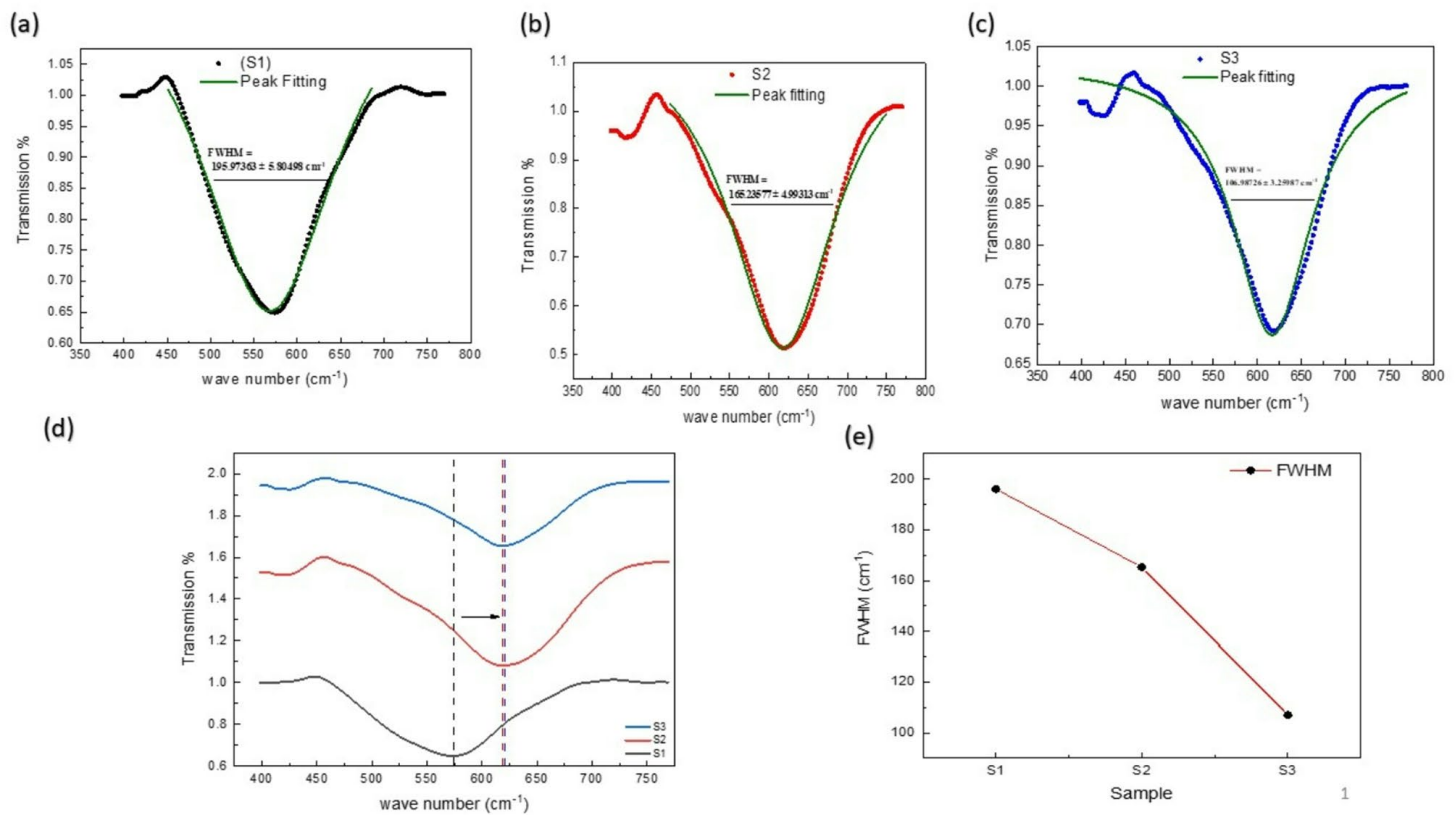
The quantitative FTIR analysis of LSMCO nanoparticles at different calcination temperature (650 °C (a), 1000 °C (b) and 1100 °C (c).

The quantitative FTIR analysis was performed using the Lorentzian fitting of the main band around 600 cm^- 1^ (Fig. 15). As indicated in the figure, there is a decrease in FWHM of the stretching Mn-O band around 600 cm^- 1^ with increasing calcination temperature^56–58^ in good agreement with XRD analysis in which the FWHM decreases as well. For more clarification, at 650 °C, the weak crystallization and mixed phase formation (Fig. 1a) broaden the FTIR absorbance band due to overlapping of vibrational modes. Increasing calcination temperature to 1000 then 1100 °C, enhances the crystal structure and decreases the FWHM of the FTIR band due to improve the Mn^4+^/Mn^3+^ bonding environment that stabilizes the perovskite structure^59–61^. The rightward shift of the Mn-O absorbance band towards higher wavenumber with increasing calcination temperature is an indication of the enhanced Mn^4+^ formation with shorter and stronger Mn-O bonds compared to Mn^3+^^59–61^.This is related to the enhanced incorporation of Sr^2+^ into the La site as indicated in XRD results. It is concluded that increasing calcination temperature enhances crystallinity and reduces lattice disorder that sharpens the FTIR absorbance. This effect is attributed to the decrease of Mn-O bond length that increases the vibrational resonance frequency.

## Conclusion

In summary, structural, morphological, compositional, electrical and molecular characteristics of La_0.6_Sr_0.4_Mn_0.8_Co_0.2_O_3_ (LSMCO) nanoparticles were investigated at different calcination temperatures (650, 1000, and 1100 °C). Rietveld refinement and FTIR analysis showed that the perovskite structure was formed even at high calcination temperatures. The detailed structural investigation revealed the presence of Jahn teller distortion, while the increase of electron bandwidth confirms the Co incorporation. The unsaturated hysteresis loop at high magnetic fields as well as the abrupt reversal of magnetization are an indicative to the superparamagnetic-like free spins. The particle agglomeration observed in the SEM analysis confirmed the presence of opposite dipole interactions. The EDAX analysis showed that the elemental ratio matched with the nominal stoichiometry.

## Data Availability

The generated datasets and/or analyzed during this study are available with the corresponding author upon reasonable request.
